# Surgical cognitive simulation improves real-world surgical performance: randomized study

**DOI:** 10.1093/bjsopen/zrab003

**Published:** 2021-05-22

**Authors:** J Cragg, F Mushtaq, N Lal, A Garnham, M Hallissey, T Graham, U Shiralkar

**Affiliations:** Department of Vascular Surgery, Russells Hall Hospital, Dudley, UK; School of Psychology, University of Leeds, Leeds, UK; Department of General Surgery, Queen Elizabeth Hospital, Birmingham, UK; Department of Vascular Surgery, New Cross Hospital, Wolverhampton, UK; Department of General Surgery, Queen Elizabeth Hospital, Birmingham, UK; Postgraduate School of Surgery, West Midlands Deanery, UK; Worcestershire Health and Care NHS trust, Worcestershire, UK

## Abstract

**Background:**

Despite the acknowledgement of human factors, application of psychological methods by surgeons to improve surgical performance is sparse. This may reflect the paucity of evidence that would help surgeons to use psychological techniques effectively. There is a need for novel approaches to see how cognitive training might be used to address these challenges.

**Methods:**

Surgical trainees were divided into intervention and control groups. The intervention group received training in surgical cognitive simulation (SCS) and was asked to apply the techniques while working in operating theatres. Both groups underwent procedure-based assessment based on the UK and Ireland Intercollegiate Surgical Curriculum Programme (ISCP) before the training and 4 months afterwards. Subjective evaluations of SCS application were obtained from the intervention group participants.

**Results:**

Among 21 participants in the study, there was a statistically significant improvement in 11 of 16 procedure-based assessment domains (*P* < 0.050) as well as a statistically significant mean reduction in time to complete the procedure in the intervention group (–15.98 *versus* –1.14 min; *P* = 0.024). Subjectively, the intervention group experienced various benefits with SCS, especially in preoperative preparedness, intraoperative focus, and overall performance.

**Conclusion:**

SCS training has a statistically significant impact in improving surgical performance. Subjective feedback suggests that surgeons are able to apply it in practice. SCS may prove a vital adjunct for skill acquisition in surgical training.

## Teaser

The aim was to address previous methodological flaws by objectively and subjectively assessing the effect of surgical cognitive simulation on both simulated and real-world surgical practice. Surgical trainees were randomized to receive training, and their simulated performance was assessed objectively using validated methods before and afterwards; the subjective effect this had on their real-world practice was also assessed. There were significant improvements in technical scores and speed following surgical cognitive simulation training, and real-world transferability over a prolonged period of time was demonstrated.

## Introduction

A key component of surgical training is the acquisition and development of technical skills. In recent years, changes in patterns of work, the expanding range of procedures, and technical demands have altered pathways of learning. The reduction in workplace experience has been identified as a training risk, and the role of simulation has been investigated as a method of preparing individuals before patient contact and developing skills^1^. Although simulation has been shown to be useful in aiding skill development^2^, there are considerable limitations in its application to surgery^3^. The qualitative experience of non-virtual reality simulators frequently falls short of expectations. Virtual reality simulators are expensive and resource-intensive, and there are doubts about transferability of the skills acquired to the operating theatre^4^^,^^5^^,^^7^^,^^8^.

The role of human factors in surgery has led to interest in the application of psychological techniques to surgical performance, reflected by more than 1000 publications on this topic in surgical journals, mostly in the past decade^9^. Many studies have shown benefits of mental practice^10^, with a meta-analysis^11^ of RCTs demonstrating enhancement in surgical skills. Although earlier studies focused on mental practice or rehearsal for improving psychomotor skills, recent studies have applied wider cognitive skills such as mental readiness and anticipatory planning. These have shown much broader benefits in surgical performance, including better stress management and situational awareness^12–17^.

In spite of favourable outcomes, the application of psychological methods in surgery remains far from commonplace, in contrast with other groups such as the military^18^^,^^19^, police^20^ or athletes^21^, who also perform under stressful conditions and have benefited demonstrably from psychological interventions. This infrequent use of psychological methods by surgeons may reflect variable methodologies in studies, and the dearth of practical relevance in the evidence concerning training, processes, and outcomes. Most studies have failed to assess the impact of psychological methods on surgical performance in real operating theatres. Skills assessed have been relatively simple or a single surgical procedure on a simulator. In some studies^22–24^, subjects were not surgeons or were assessed for short-term changes.

This pragmatic study set out to address these methodological issues by linking subjective operating theatre assessment with objective assessment in the skills laboratory. A training programme, surgical cognitive simulation (SCS), was designed for application in operating theatres. SCS comprised evidence-based performance-enhancing strategies, including preperformance routines, goal setting, thought management and focused, mental practice. A previous study^26^ had examined the feasibility of providing SCS training for surgical trainees, and the aim of the present work was to assess the real-life efficacy of SCS.

A prospective RCT was therefore designed to see whether SCS training delivered to surgical trainees led to the application of these skills in clinical situations. A further objective was to determine whether such application of SCS improved surgical skills in the laboratory and overall surgical performance in the operating theatre.

## Methods

This RCT examined the effect of SCS on a simulated operative procedure and subjective experiences while performing in the operating theatre. The School of Psychology Research Ethics Committee at the University of Leeds granted ethical approval (reference 17-0166).

General surgical trainees in years 3–5 from the West Midlands Deanery were recruited and consented to participate. Baseline details for each candidate were collected via questionnaires to assess level of surgical experience. Participation was voluntary and candidates could withdraw at any point. Participants were randomized by means of a random number generator to either an intervention group that received SCS training or a control group that received no SCS training.

Frozen and thawed porcine specimens that provided acceptable tissue handling with dissection were used. A baseline video recording was made of all participants performing laparoscopic cholecystectomy on an *ex vivo* model. Instrument availability was kept constant, as was the seniority of the surgical assistant. Videos were graded objectively by assessing surgeons in a blinded fashion according to the Intercollegiate Surgical Curriculum Programme problem-based assessment (PBA) criteria for laparoscopic cholecystectomy, a validated objective assessment tool used for all UK surgical trainees in relation to operative training. Multiple assessors were used to minimize inter-rater variation.

SCS training (*Table S1*) was provided for the intervention group by a surgical performance coach along with reading material on SCS for self-study. The participants were advised to perform cognitive simulation for approximately 20 min on the day before undertaking procedures.

At 4 months after the initial assessment, all candidates underwent another video assessment of laparoscopic cholecystectomy performed on porcine specimens. Those in the intervention group provided subjective information about their experience of practising and applying cognitive simulation in their work (*Table S2*).

Sixteen domains from the PBA for laparoscopic cholecystectomy were considered relevant to a simulated operation, in addition to time taken to complete the procedure and a global performance score, for which the principles of the PBA terms were used; results were converted to a numerical score ranging from 1 (poor) to 10 (excellent). The mean rating for each dependent variable was taken, and these were used to compare performance across the two groups. For each group, a difference score was obtained by subtracting the performance score (mean score provided by assessors) at baseline from that at the end of training.

### Statistical analysis

Previous studies suggested that nine participants per group would be required to show statistical significance at *P* < 0.050 with 80 per cent power^26^, so 21 trainees were used, 10 assigned to the intervention and 11 to the control group. Video assessors were blinded to which group the candidate was assigned, but not to whether the video showed a baseline or follow-up attempt.

Objective scores from each assessor were assessed for κ correlation. Before undertaking the inferential analysis, the Shapiro–Wilk test was used to check for normality in the distribution of the data for each dependent variable. Of the 18 (16 from ISCP) domains, three had statistically significant scores indicating that they violated the assumptions of normality. For these measures, the non-parametric Wilcoxon rank-sum test was used to compare differences between the two groups, and an independent two-sample Student’s *t* test was employed for the remaining 15 variables. A false discovery rate correction was used to control for the increase in type I errors in null hypothesis testing that arise when conducting multiple comparisons; this was done by ranking all *P* values in ascending order and applying the formula (i/m)Q, where i is the rank of the individual *P* value, m is the total number of tests, and Q the false discovery rate. The false discovery rate was fixed at 0.050 for this study, setting the expectation that 5 per cent of reported results would be false-positives. This correction was implemented using the p.adjust function from the stats package in R version 3.1.2 (R Foundation for Statistical Computing, Vienna, Austria)^27^.

## Results

**Fig. 1:**
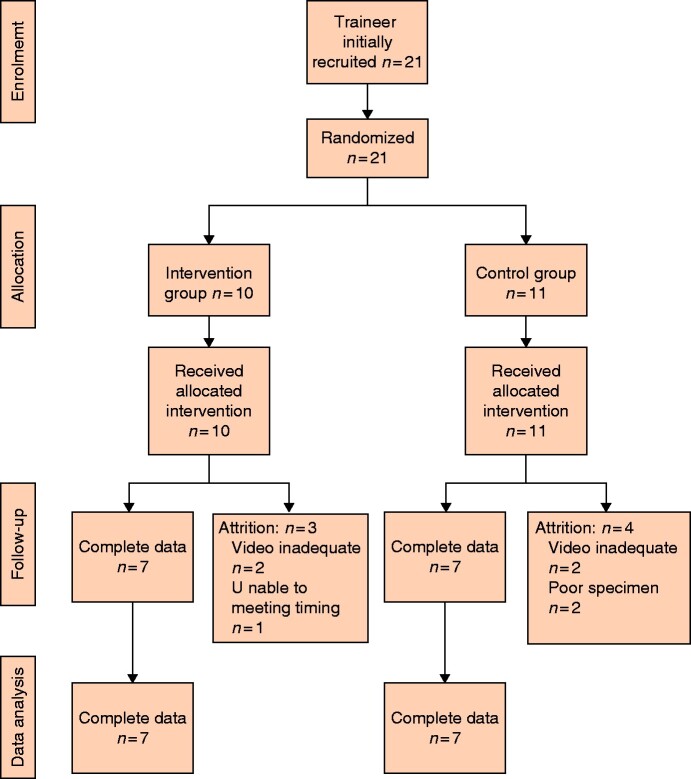
Study flow diagram

Of 21 trainees recruited to the study, all performed the procedures, but recordings from seven trainees were not available for assessment for technical reasons. Complete objective data were available for seven trainees in the intervention group and seven controls (*Fig. 1*). Two expert surgeons with 15 years of experience rated the video recordings. The assessors had a statistically significant moderate level of agreement (κ  =  0.434; z = 3.55; *P* < 0.001).

There was no significant difference at baseline in terms of logbook experience of laparoscopic cholecystectomy, with a mean total of 101 procedures for trainees in the control group control and 108 in the intervention group (*P* = 0.425) (*Table 1*). There was no significant difference between the perceived adequacy of the specimens or video between the groups (6.357 (control) *versus* 7.142 (intervention); *P* = 0.111).

**Table 1:** Participant characteristics

	Intervention (*n* = 7)	Control (*n* = 7)
Age (years)*	35.4	32.7
**Sex ratio (M : F)**	5 : 2	6 : 1
Postgraduate years*	10	7.3
No. of laparoscopic cholecystectomies*	108	101
Assisted	68.9	101
Trainer scrubbed	32.3	54
Trainer unscrubbed	4	5
Performed	3.3	14

* Values are mean.

**Fig. 2:**
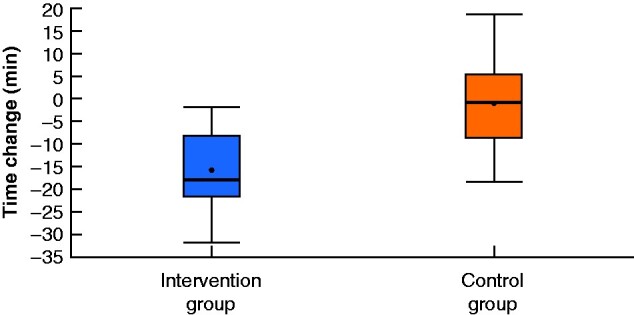
Mean change in time taken to complete the procedure in intervention and control groups Median (bold line), mean (dot), interquartile range (box), and range (error bars) are shown. *P*=0.024 (independent T- test).

**Fig. 3:**
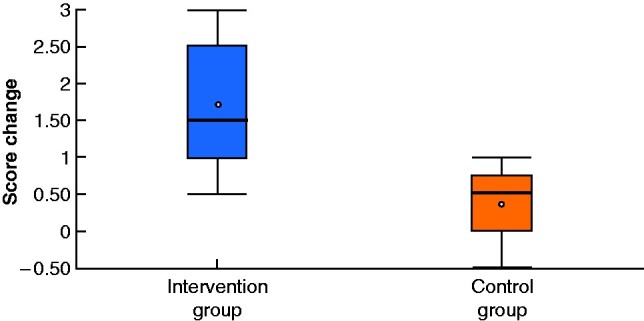
**Mean change in global performance score in intervention and control groups** Median (bold line), mean (dot), interquartile range (box), and range (error bars) are shown. *P*=0.011 (independent T-test).

**Fig. 4:**
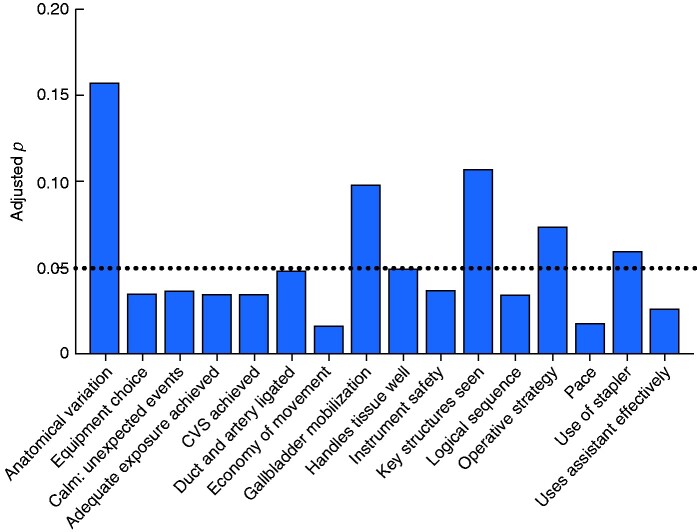
Significance of differences in mean change in probem-based assessment scores between intervention and control groups CVS, critical view of safety. Dotted line represents cut-off for significance (*P* < 0.050, Wilcoxon rank sum).

A greater improvement was observed in the intervention group in terms of procedure times (**–**15.98 min in intervention group *versus* –1.14 min in control group; *P* = 0.024) (*Fig. 2*), global performance scores (+1.742 *versus* +0.357; *P* = 0.011) (*Fig. 3* and *Table 2*), and in 11 of 16 individual scoring domains (*Fig. 4*). Subjectively, SCS was well received and there was perceived value in its application to real-world situations. Trainees reported a greater sense of preoperative preparedness and intraoperative focus in addition to feeling an overall improvement in their quality of performance (*Table 3*). Many (88 per cent) expressed a view that SCS should be incorporated into the training programme.

**Table 2:** Effect size of surgical cognitive simulation on each domain

	Mean difference	Cohen’s d
Time (min)	1.071 (3.819) (–6.779, 8.921)	0.1060
Operative strategy score	0.321 (0.480) (–0.665, 1.308)	0.2531
Equipment choice score	0.000 (0.330) (–0.679, 0.679)	0.0000
Adequate exposure achieved score	0.750 (0.554) (–0.389, 1.889)	0.5116
Key structures seen score	1.143 (0.625) –0.141, 2.427)	0.6914
Logical sequence score	0.107 (0.417) (–0.749, 0.964)	0.0972
Handles tissue well score	0.607 (0.643) (–0.714, 1.929)	0.3569
Use of stapler score	0.643 (0.370) (–0.118, 1.404)	0.6561
Instrument safety score	0.179 (0.447) (–0.741, 1.098)	0.1508
Pace score	0.214 (0.617) (–1.055, 1.484)	0.1312
Economy of movement score	0.250 (0.630) (–1.046, 1.546)	0.1499
Responds to anatomical variation score	0.321 (0.422) (–0.546, 1.189)	0.2877
Deals calmly with unexpected events score	0.393 (0.284) (–0.191, 0.976)	0.5232
Uses assistant effectively score	0.393 (0.441) (–0.514, 1.300)	0.3365
Critical view of safety achieved score	0.607 (0.767) (–0.970, 2.184)	0.2992
Duct and artery ligated score	0.857 (0.481) (–0.131, 1.845)	0.6741
Gallbladder mobilization score	0.571 (0.670) (–0.806, 1.949)	0.3223
Overall score	0.357 (0.637) (–0.952, 1.667)	0.2119

Values are mean(s.e.m.) (95 per cent c.i.).

**Table 3:** Subjective feedback

	Weighted score
Improved sense of preparedness	7.9
Improved confidence	7.4
Improvement in time taken to perform procedure	6.7
Improvement in quality of focus while operating	7.6
Improvement in ability to learn while assisting	7.5
Improvement in decision-making while performing a procedure	6.2
Improvement in team working skills during operative procedure	5.56
Improvement in performing new technique or approach	7.4
Improvement in overall quality of surgical performance	7.5
Improvement in dealing with unexpected situations in routine procedure	6.1
Improvement in operating on emergencies	6.3
Improvement in stress of performing operations	7.2
Improvement in feeling of fatigue after operating list	6.11
Do you see any role of cognitive simulation in surgical training? (yes/no)	88% yes

Weighted mean scores of subjective improvement experienced in specific domains in the intervention group are shown. Score range 0–10, with 10 representing most and 0 least perceived improvement.

## Discussion

This study has demonstrated successful application of psychological techniques by surgeons in a clinical environment and its effect on performance in real life. The results indicate that SCS training helped trainees to practise independently and apply it in operating theatres. Application of SCS showed objective improvement in surgical skills on a simulated procedure, and subjective improvement in various performance domains in the operating theatre.

Aside from physical operative experience, evidence suggests that non-operative skills can independently improve performance and outcomes in surgery^28^. At the same time, fatigue, memory failures, and lapses in concentration are common reasons for patients coming to harm. Lack of cognitive skills has been identified as a common contributor to this^29^. The present study mirrors other findings that psychological methods improve surgical skills along with surgeons’ mental well-being^30^. This is an important aspect when considering the growing recognition of stress and burnout among surgeons^31^.

The present study has overcome some of the methodological limitations in other studies of skill improvement^32^, including the use of surgical trainees as subjects instead of medical students^33^^,^^34^, applicability to more than one procedure^36^, evaluation of both simulated and real procedures^35^, as well as assessing long-term effects in the operating theatre^36^. It has demonstrated durability of this approach with delayed testing after unsupervised practice, representing a more realistic approach for implementation of psychological methods into training^6^^,^^10^^,^^37^.

The term SCS merits explanation. In a recently published review, the authors concluded that establishing a taxonomy for mental skills in surgery would help in the development of robust mental skills training programmes to promote optimal surgeon wellness and performance. The lack of use of psychological methods in surgical practice is thought to be due to wide variation in study methodologies, lack of clarity about processes, and use of imprecise terminology such as ‘mental practice’, ‘mental rehearsal’ or ‘mental imagery’. Terms such as mental imagery and visualization are not only imprecise, but also counterproductive, as they limit application of the cognitive process to visual information. Unless multiple sensory modalities are applied, the performer is unlikely to experience the benefit of cognitive simulation. Mental practice usually covers very limited psychomotor functions, but surgical skill acquisition also requires situational awareness, anticipation, and other cognitive functions that are addressed in SCS. Thus, SCS is not just a different label for an established term such as mental practice, but a reflection of a distinct cognitive process with a new perspective.

An earlier study^25^ had shown that SCS training was well received by trainees, although follow-up after the initial training revealed that application of SCS dropped significantly after 4 weeks. In the present study, participants were asked every month to provide details about their application of SCS in the previous 4 weeks. From the feedback, it was possible to grade the quality of cognitive simulation of each participant. A correlation has been shown between quality of imagery and performance improvement^38^. The present results agree with other studies showing that surgical performance improved proportionately with the quality of mental imagery scores^10^.

There are some limitations to this study. The sample size was very small and data were lost for seven subjects. Considering the outcomes, however, it seems unlikely that additional data from three intervention and four control subjects would have substantially altered the results. Inclusion of a subjective evaluation can be seen as another limitation. However, the objective was to conduct a pragmatic study with practical relevance. This would not be the case if the study had omitted evaluations during routine working in the operating theatre. Simulated assessments have been shown to offer a reasonably good reflection of performance in the real world^39^. In addition, as SCS is a subjective process, subjective assessment needed to be considered. A Hawthorne effect—the temporary alteration of behaviour by the subjects of a study owing to their awareness of being observed—cannot be discounted^39^.

The results of this study have implications for the manner in which surgeons acquire and improve surgical skills. Although both the Chief Medical Officer in the UK^40^ and the American College of Surgeons^41^ have suggested simulation technology as a training strategy, the present results suggest that SCS may be an effective adjunct to technical simulation. SCS is designed to help surgeons to focus on their sensory capabilities and simulate the procedure before undertaking it. The fundamentals of SCS are underpinned by existing psychological and neuroscience theories^42^. By mentally practising the procedure, surgeons may need to spend less time in the skills laboratory, shortening their learning curve by using the ‘simulation centre in the brain’. It is free, available to all, and much less resource-intensive than other approaches to training. After initial training with a facilitator, trainees can perform it anywhere and in their own time. Those responsible for surgical training need to acknowledge the benefit of such training and incorporate it as an essential part of surgical training.

## Funding

The project team received funding from Health Education England (West Midlands). F.M. was supported by a Fellowship from the Alan Turing Institute and an MRC Confidence in Concept Award (MC/PC/17165).

*Disclosure.* The authors declare no conflict of interest.

## Supplementary material

Supplementary material is available at *BJS Open* online.

## Supplementary Material

zrab003_Supplementary_Data
