# Supplementary material for: Surgical cognitive simulation improves real-world surgical performance: randomized study
Source: BJS Open. 2021 May 22;5(3):zrab003. doi: 10.1093/bjsopen/zrab003 (PMC8140200; doi:10.1093/bjsopen/zrab003)
Supplement: zrab003_Supplementary_Data [file zrab003_supplementary_data.zip › Supporting information.docx]

**Supporting information Table S1 – Surgical Cognitive Simulation Programme**

‘Surgical Cognitive Simulation’ training programme

Initial group training took place for 3 hours. During which participants were given information about the role of sensory modalities in surgical skill acquisition. Training was provided to develop ‘cognitive simulation’ by generating multiple sensory modalities especially visual, tactile, kinaesthetic and verbal.

A practical schedule for practising the technique at the subjects’ own pace was given along with the reading material for self-study.

A multi-sensory self-evaluation form was designed to monitor the quality of simulation.

Participants were advised to return the completed form to the performance coach after first month.

After the 2^nd^ month a 30-minute telephone discussion was conducted with each participant. Advice was given where required to maintain the implementation of SCS.

At the end of 3^rd^ month, participants were asked to provide a ‘SCS’ script for a surgical procedure they had performed. The quality of cognitive simulation was rated according the contents of the script.

At the end of 4^th^ month participants were asked to provide responses to the questionnaire depicting their experience of applying SCS in the course of clinical work.

**Supporting information, Table S2 - Subjective feedback**

**Self-monitoring form**

Number - Date: _____________________________

If you have not practiced a particular modality for any reason, you need not rate the specific modality and can leave it blank

**A) Visual modality**

1) **Resolution (Quality of the picture)**

0 1 2 3 4 5 6 7 8 9 10

Poor Excellent

2) **Dimensions (Anatomical relationships, 3D perceptions)**

0 1 2 3 4 5 6 7 8 9 10

Poor Excellent

3) **Timing (Speed of the actions)**

0 1 2 3 4 5 6 7 8 9 10

Poor Excellent

4) **Control (Internal/external visualisation, change in the speed)**

0 1 2 3 4 5 6 7 8 9 10

Poor Excellent

B) **Tactile modality**

0 1 2 3 4 5 6 7 8 9 10

Poor Excellent

C) **Kinaesthetic modality**

0 1 2 3 4 5 6 7 8 9 10

Poor Excellent

D) **Auditory modality**

0 1 2 3 4 5 6 7 8 9 10

Poor Excellent

E) **Self-talk/verbal**

0 1 2 3 4 5 6 7 8 9 10

Poor Excellent
